# Increased biological activity of protein Kinase C gamma is not required in Spinocerebellar ataxia 14

**DOI:** 10.1186/s13041-017-0313-z

**Published:** 2017-07-24

**Authors:** Etsuko Shimobayashi, Josef P. Kapfhammer

**Affiliations:** Anatomical Institute, Department of Biomedicine Basel, University of Basel, Pestalozzistrasse 20, 4056 Basel, Switzerland

**Keywords:** Spinocerebellar ataxia type 14, Protein kinase C gamma, Purkinje cell dendritic development

## Abstract

Spinocerebellar ataxia (SCA) is an autosomal dominant neurodegenerative disorder characterized by slowly progressive cerebellar dysfunction. Currently, 42 SCA types are known, some of which are caused by CAG repeat expansions, but others are caused by point mutations or deletions. Spinocerebellar ataxia type 14 (SCA14) is caused by missense mutations or deletions in the *PRKCG* gene, coding for protein kinase C gamma (PKCγ). It is still not well understood how these mutations eventually cause Purkinje cell dysfunction and death. Because PKCγ is a well characterized signaling protein highly expressed in Purkinje cells SCA14 offers the chance to better understand the pathogenesis of Purkinje cell dysfunction and death. Altered biological activity of PKCγ would be the simplest explanation for the disease phenotype. There are indeed indications that the enzymatic activity of mutated PKCγ proteins could be changed. Many mutations found in SCA14 families are located in the regulatory C1B and C1A domain, while a few mutations are also found in the C2 and in the catalytic C3 and C4 domains. For many of these mutations an increased enzymatic activity could be demonstrated in cell-based assays, but it remains unclear whether there is indeed an altered biological activity of the mutated PKCγ proteins within living Purkinje cells. In this study we used the dendritic morphology of developing Purkinje cells to detect increased biological activity of PKCγ after expression of different mutated PKCγ proteins. Our results indicate that two out of three known mutations in the catalytic domain of PKCγ did indeed show increased biological activity. On the other hand, none of the five tested mutations located in the regulatory C1 or the C2 domain showed an increased biological activity. Our findings indicate that SCA14 mutations located in different domains of the *PRKCG* gene cause SCA14 by different mechanisms and that an increased constitutive activity of PKCγ may be one, but cannot be the only mechanism to cause disease in SCA14.

## Introduction

Spinocerebellar ataxias (SCAs) are a group of inherited diseases with mutations in diverse genes, the most common forms are caused by Poly-glutamine repeats in the respective genes. Some forms are also caused by point mutations in particular genes, and in SCA14 such mutations are found in the *PRKCG* gene [1], which is coding for protein kinase C gamma (PKCγ) [2]. SCA14 is inherited in an autosomal dominant fashion and clinically characterized by a slowly progressive cerebellar ataxia accompanied by degeneration of Purkinje cells [3]. Protein kinase C (PKC) is a family of serine/threonine kinases that are important signaling molecules in many cells [4]. The γ isoform of PKC (PKCγ) belongs to the classical PKC family and the protein has four conserved domains and five variable regions. Activation of PKCγ is controlled by the two regulatory domains, with diacylglycerol (DAG) and phorbol esters binding to the C1 domain and Ca^2+^ binding to the C2 domain [5]. The catalytic domain is formed by the C3 region which contains an ATP binding site and the C4 region which constitutes the main catalytic site and which is strongly conserved among PKC isoforms and also among different species. The majority of mutations found in SCA14 families are located in the regulatory C1B and C1A domain, while a few mutations are also found in the C2 and in the catalytic C3 and C4 domain [6]. It is still not clear how the different mutations may cause SCA14. It is unlikely to be a simple loss of function mechanism, because this would not be easily compatible with the autosomal dominant inheritance and PKCγ knockout mice only show mild ataxia and no morphological abnormalities in the cerebellum [7, 8].

Activity of PKCγ is also an important determinant of Purkinje cell dendritic development. In cerebellar slice cultures, activation of PKC causes a strong inhibition of Purkinje cell dendritic growth and development, on the other hand, inhibition of PKC activity promoted Purkinje cell dendritic growth [9]. The morphology of developing Purkinje cells thus might be used as an indicator of the endogenous activation of PKCγ in Purkinje cells because a strong or constitutive activation will result in Purkinje cells with strongly reduced dendrites. The dendritic alterations of Purkinje cells found after PKC activation would also be compatible with the hypothesis that pathogenesis of SCA14 might be due at least in part to a gain of function of PKCγ with too strong or constitutive activation of PKC activity. This hypothesis is also supported by findings that many PKCγ mutations have an increased PKCγ catalytic activity when tested in in vitro or in cell based assays [6, 10, 11]. On the other hand, there are also findings which showed that C1B domain mutations of PKCγ might be functionally defective due to decreased binding to DAG [12]. It is also suggested that SCA14 mutations may alter the structure of PKCγ making it unstable or favoring aggregate formation, which eventually might cause loss of PKCγ function [13, 14]. However in Purkinje cells transfected with mutant PKCγ, abnormal dendritic development has been seen irrespective of aggregate formation [15]. Taken together, it is still unclear whether the high kinase activity found in cell based assays will eventually lead to increased biologically activity of PKCγ in Purkinje cells and may contribute to the pathogenesis of SCA14.

We have previously reported that transgenic mice carrying a PKCγ mutation in the catalytic domain (S361G) have pathological changes and motor deficits typical for cerebellar ataxias [16]. In organotypic slice cultures, Purkinje cells from PKCγ-S361G transgenic mice showed severe inhibition of dendritic development which was identical to Purkinje cells treated with a PKC activator [16]. These findings show that PKCγ-S361G has increased biological PKC activity within Purkinje cells and raise the possibility that the increase in PKC activity is an important determinant of pathogenesis in SCA. We have now addressed the question whether a similar increase in biological activity of PKCγ (identified by changes in Purkinje cell dendritic development) might also be present in other mutations known to cause SCA14. In order to address this question, we have investigated the morphology of Purkinje cells in cerebellar slice cultures from three transgenic mouse lines which carry mutations in different domains of PKCγ. Moreover, we have tested the effect of these and additional SCA14 mutations on Purkinje cell dendritic development in dissociated cerebellar cultures after transfection with an L7 based expression plasmid [17, 18]. Our findings indicate that two SCA14 mutations located in the catalytic domain, but not in other domains, result in an increased PKC activity with dendritic changes of Purkinje cells. These findings suggest that SCA14 mutations located in different domains of the *PRKCG* gene might cause SCA14 by different mechanisms and that an increased constitutive activity of PKCγ may be one, but is unlikely to be the only mechanism to cause disease in SCA14.

## Methods

### Generation of transgenic mice

Animal experiments were carried out in accordance with the EU Directive 2010/63/EU for animal experiments and were reviewed and permitted by Swiss cantonal authorities. The transgenic experiments were done in a FVB background at the Transgenic Animal Facility of the Biozentrum, University of Basel, using the pronuclear microinjection method. To identify founders, genotyping with genomic DNA samples from biopsies was performed by PCR. The primers for first genotyping were: forward primer 1, 5′-gacccctccagaccgcctag tcctg-3′ and reverse primer 1, 5′-gcctatggaaaaacgccagcaacgc-3′; for second genotyping a 585 bp band can detected with specific primers: forward primer 2, 5′-gagacttgatgtaccacattcaacag-3′ and reverse primer 2, 5′-ggcggggtctgaaaggaggcggg-3′. Then the transgenic human PKCγ gene was confirmed by DNA sequencing. The fragment for sequencing was obtained by PCR with genomic DNA samples and primers (forward primer, 5′-gtcgagtttactccctatcagtgatag-3′ and reverse primer, 5′-tagtcctgtcgggtttcgccacctc-3′). The confirmed transgenic founders were crossed with FVB-Tg (Pcp2-tTA) 3 Horr/J transgenic mice (Jackson Laboratory, Sacramento, CA, USA) to generate Pcp2-tTA/TRE-PKCγ double transgenic mice. For Pcp2-tTA genotyping a 472 bp band can detected with specific primers: forward primer, 5′-gcgctgtggggcattttactttagg-3′ and reverse primer, 5′-caacatgtccagatcgaaatcgtc-3′.

### Plasmid construction

Human *PRKCG* gene was from Origene in pCMV6-XL4 (Rockville, MD, USA). Mutated or deleted *PRKCG* genes were produced by site-directed mutagenesis PCR with the pCMV6-XL4-*PRKCG*. PCR was performed with the Pfu DNA polymerase (Invitrogen, Carlsbad, CA, USA) for 30 cycles (30 s at 95 °C, 45 s at 57 °C, and 8 min at 68 °C), using the following primers:

- G118D forward primer: 5′-ctgcgaccactgtgactccctcctctacgggctt-3′, reverse primer: 5′-aaggtggggctgctgtagctatgca-3′;
- S119P forward primer: 5′- ttctgcgaccactgtggcttcctcctctacgggcttgt-3′, reverse primer: 5′- acaagcccgtagaggaggaagccacagtggtcgcagaa-3′;
- V138E forward primer: 5′- cctgctgcgagatgaacgagcaccggcgctgtgtgcgt-3′, reverse primer: 5′- acgcacacagcgccggtgctcgttcatctcgcagcagg-3′;
- I173S forward primer: 5′-ctcccacagcagatgagagccacgtaactgttggcg-3′, reverse primer: 5′-cgccaacagttacgtggctctcatctgctgtgggag-3′;
- Deletion 260–280 forward primer: 5′-gaggagggcgagtattacaatgtgc-3′, reverse primer: 5′-catggcccccatgaagtcgttgcg-3′;
- G360S forward primer: 5′-cat ggttctaggaaaaagcagttttgggaaggtga-3′, reverse primer: 5′-tcaccttcccaaaactgctttttcctagaaccatgagg-3′;
- S361G forward primer: 5′-gttctaggaaaaggcggttttgggaaggtgatgctg-3′, reverse primer: 5′-catgaggaagctgaagtcggagatg-3′;
- F643 L forward primer: 5′- gcagcggcgagaacttagacaagttcttcacgc-3′, reverse primer: 5′- gcgtgaagaacttgtctaagttctcgccgctgc-3′.

The PCR products were then incubated with DpnI which only digests the parental methylated cDNA and the constructed mutated expression vectors were confirmed by DNA sequencing (Microsynth, Balgach, Switzerland). These PCR products fragments and L7-GFP vector were incubated with restriction enzymes, Not1 and Nco1 (New England BioLabs, Massachusetts, USA) in suitable buffer. After 30 min incubation in a 37 °C water bath, DNA fragment and linearized vector were fused using T4 ligase (New England BioLabs, Massachusetts, USA). The constructed expression vectors were confirmed by DNA sequencing (Microsynth, Balgach, Switzerland).

### Quantitative real-time PCR

For gene expression analysis, quantitative real-time PCR reactions were conducted in a total volume of 20 μl comprising 10 μl of Mastermix with SYBR green (Applied biosystems), 0.5 μl of each primer (1.0 μM), 0.3 μl of sample cDNA, and 8.5 μl ultrapure water. Real-time PCR reactions were run on a 48-well format with a One step real-time detector (Applied biosystems) under the following reaction conditions: 1 cycle of (10 min at 95 °C), 40 cycles of (15 s at 95 °C and 60 s at 65 °C), and 1 cycle of (15 s at 95 °C, 30 s at 72 °C and 15 s at 95 °C). The following primers were used:

- Human *PRKCG*
  (forward: cacgaagtcaagagccaca, reverse: tagctatgcaggcggaactt)
- Mouse *Prkcg*
  (forward:, cacgaggtgaagagccacaa, reverse: tagctgtgcagacggaactt)
- *Gapdh*
  (forward: aactttggcattgtggaagg, reverse: acacattgggggtaggaaca)

Oligonucleotide primers were designed using the Primer 3 software (http://bioinfo.ut.ee/primer3//). As endogenous control Gapdh was selected. Reactions were quantified by the relative standard curve system and the cycle threshold (Ct) method using the SDS2.2 software (Applied Biosystems, Foster City, CA). The relative quantitation value (RQ) for each sample with triplicates was acquired from two independent experiments and was calculated for each gene. The data were analyzed with Graph Pad Prism software.

### Organotypic slice cultures

Animal experiments were carried out in accordance with the EU Directive 2010/63/EU for animal experiments and were reviewed and permitted by Swiss cantonal authorities. Cultures were prepared from B6CF1 (CB6) mice or mutant PKCγ transgenic mice [16] as described previously [19]. Briefly, mice were decapitated at postnatal day 8 (P8), their brains were aseptically removed and the cerebellum was dissected in ice-cold preparation medium (minimal essential medium (MEM) with 1% glutamax (Life Technologies, Zug, Switzerland), pH 7.3). Sagittal sections 350 μm thick were cut on a McIllwain tissue chopper under aseptic conditions. Slices were separated, transferred onto permeable membranes (Millicell-CM, Merck-Millipore, Zug Switzerland) and incubated on a layer of Neurobasal medium (97% Neurobasal medium, 2% B27 supplement, 1% glutamax, pH 7.3) in a humidified atmosphere with 5% CO2 at 37 °C. The medium was changed every 2–3 days for a total of 5 days for microarray analysis and of 7 days for protein analysis and immunostaining.

### Dissociated cerebellar cultures

Dissociated cerebellar cultures were prepared from mice essentially as described [17, 18]. Briefly, mice were decapitated at P0, Cerebellums were removed and transferred into a sterile 35 mm tissue culture dish that was kept on ice and filled with ice-cold, modified Hank’s Balanced Salt Solution (HBSS) (MHS; 5.333 mM KCl, 0.441 KH_2_PO_4_, 137.931 mM NaCl, 0.336 mM Na_2_HPO_4_-7H_2_O, and 5.556 mM D-glucose) (GIBCO, Invitrogen). After that, the isolated cerebellar primordia were minced using a scalpel to obtain chunks. Subsequently, the cerebellar tissues were digested by adding 250 μl of freshly prepared, ice-cold papain solution (MHS containing 20 U/ml papain, (Warthington, CA, USA)) to each tube, followed by incubation in a 34 °C for 30 min. To stop the digestion, 1 ml MHS/FBS (84% *v*/v MHS, 16% *v*/v fetal bovine serum) was added to each tube. After gentle mixing by inverting the tube, the cells were harvested by centrifugation at RT for 4 min at 0.6×g. All subsequent steps were carried out at RT under the hood. After all supernatants were removed, 350 μl of freshly-prepared DNase I solution (MHS containing 11.86 mM MgSO_4_ and 5 U/ml DNase I) was added to each of the harvested cerebellar cell pellets. Each cell pellet was then triturated carefully by pipetting up and down with a sterile 1000 μl tip. The triturated cells were then passed through a 180 μm nylon mesh (Millipore, Zug Switzerland), collected in a fresh 1.5 ml tube, and harvested by centrifugation (RT for 4 min at 0.6×g). The cells were then washed twice with MHS. Cells were re-suspended with plating medium containing 90% *v*/v DFM, 1% N-2 Supplement (GIBCO, Invitrogen), 1% Glutamax (GIBCO, Invitrogen) and 10% *v*/v FBS (GIBCO, Invitrogen), pH were adjusted to 7.2–7.4 and plated in glass chamber that had been coated poly-L-lysine. 2 h after transfection, 0.4 ml DFM, containing 1% N-2 Supplement, 1% Glutamax at 37 °C was added to each well. After that, half of medium were changed once or twice a week. For PKC activation, 15 nM Phorbol 12-myristate 13-acetate (PMA) (Tocris, Bioscience, United Kingdom) was added to the medium at each change for a total of 7 days, starting at DIV4 or DIV7.

### Plasmid transfection to dissociated cerebellar cultures

The transfection of the cells was performed using the mouse neuron Nucleofector kit (Lonza, Switzerland) according to manufacturer’s instructions. 100 μl of the Nucleofection solution (Lonza, Switzerland) was mixed with the plasmid DNA to be transfected. This mixture was then used to suspend the cerebellar cell pellet. Cell suspension was transferred into one of the cuvettes provided in the kit and subjected to an optimized program. After transfection, cells were plated in a glass chamber that had been coated with poly-L-lysine containing 90% *v*/v DFM, 1% N-2 Supplement (GIBCO, Invitrogen), 1% Glutamax (GIBCO, Invitrogen) and 10% *v*/v FBS (GIBCO, Invitrogen), pH was adjusted to 7.2–7.4. 2 h after transfection, the medium was changed to non-serum conditions and half of the medium was changed once or twice a week.

### Immunohistochemistry of cerebellar slices

After 7 days, slices were fixed in 4% paraformaldehyde overnight at 4 °C. All reagents were diluted in 100 mM phosphate buffer (PB), pH 7.3. Slices were incubated in blocking solution (0.5% Triton X-100, 3% normal goat serum) for 1 h at room temperature. Two different primary antibodies were simultaneously added to the slices in fresh blocking solution and incubated overnight at 4 °C. After washing in PB, secondary antibodies were added to the slices in PB containing 0.1% Triton X-100 for 2 h at room temperature. For the analysis of protein expression in Purkinje cells, mouse anti-Calbindin D-28 K (Swant, Marly, Switzerland 1:1000) and polyclonal rabbit anti-GFP (Abcam, Cambridge, UK 1:1000) were used as primary antibodies and goat anti-mouse Alexa 568 (Molecular Probes, Eugene, OR, 1:1000) and goat anti-rabbit Alexa 488 (Molecular Probes, Eugene, OR, 1:1000) were used as secondary antibodies to visualize Purkinje cells as described before [19]. Stained slices were mounted on cover slips with Mowiol (SigmaAldrich, Buchs, Switzerland). Cultures were viewed on an Olympus AX-70 microscope equipped with a Spot digital camera. Recorded images were adjusted for brightness and contrast with Photoshop image processing software.

### Immunohistochemistry of dissociated cerebellar cells

After 14–18 days, cells were fixed in 4% paraformaldehyde for 1 h at 4 °C. All reagents were diluted in 100 mM PB, pH 7.3. Cells were incubated in blocking solution (0.5% Triton X-100, 3% normal goat serum) for 30 min at room temperature. Two different primary antibodies were simultaneously added to the cells in fresh blocking solution and incubated for 30 min at room temperature. After washing in PB, secondary antibodies were added to the slices in PB containing 0.1% Triton X-100 for 30 min at room temperature. For the analysis of vector expression in Purkinje cells, mouse anti-Calbindin D-28 K (Swant, Marly, Switzerland 1:1000) and polyclonal rabbit anti-GFP (Abcam, Cambridge, UK 1:1000) were used as primary antibodies and goat anti-mouse Alexa 568 (Molecular Probes, Eugene, OR, 1:1000) and goat anti-rabbit Alexa 488 (Molecular Probes, Eugene, OR, 1:1000) were used as secondary antibodies to visualize Purkinje cells [17]. Stained cells were viewed on an Olympus AX-70 microscope equipped with a Spot digital camera. Recorded images were adjusted for brightness and contrast with Photoshop image processing software. Transfected Purkinje cells were identified by double immunostaining for calbindin and GFP (red and green) and only double positive cells were used for statistical analysis.

### Statistical analysis

The quantification of Purkinje cell dendritic tree size was done as previously described [20]. Purkinje cells which had a dendritic tree isolated from its surroundings were selected for analysis. Cells were photographed with a digital camera (Spot Insight, Diagnostic Instruments, USA). An image analysis program (ImageJ) was used to trace the outline of the Purkinje cell dendritic trees yielding the area covered by the dendritic tree. Over 20 cells were acquired from the same experiments and analyzed using GraphPad Prism software (San Diego, USA). At least three independent experiments were acquired. The statistical significance of differences in parameters was assessed by non-parametric Mann-Whitney’s test. Confidence intervals were 95%, statistical significance when *p* < 0.05. In the experiments shown in Figs. 4 and 5 the Purkinje cells transfected with the PKCγ -WT plasmid were used as control and set as 100%.

## Results

### Transgenic mice carrying the PKCγ S361G mutation but not the G118D mutation or a 260–280 deletion showed inhibition of dendritic development in organotypic slice cultures

**Fig. 1:**
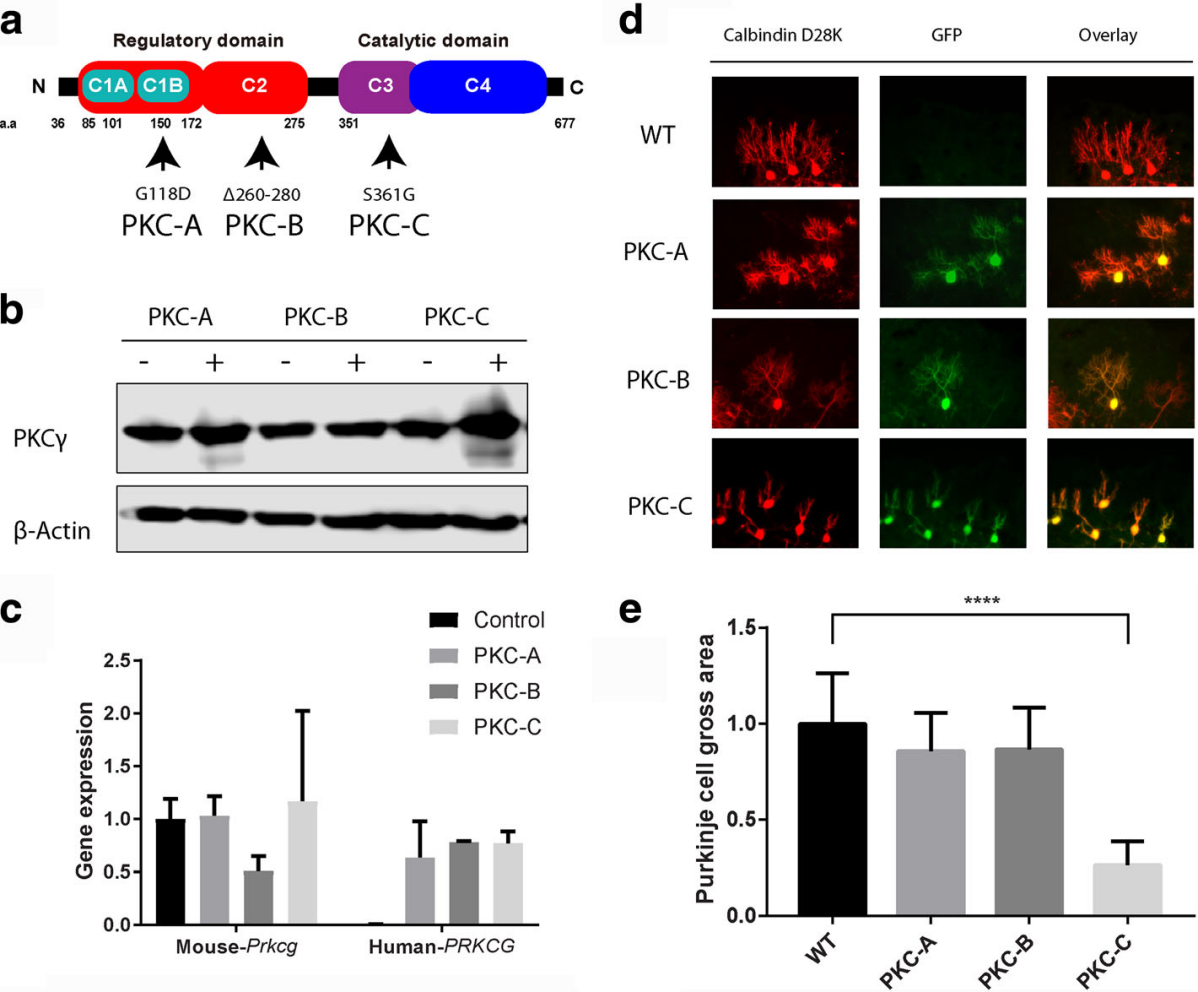
Three transgenic mice with different PKCγ mutations. **a** We generated three different PKCγ mutated transgenic mouse lines (named PKC-A, PKC-B and PKC-C). The genotype of the founders was confirmed by PCR. **b** Western blot analysis of total PKCγ protein in the cerebellum from each transgenic mouse. (−) Non transgenic mice, (+) Transgenic mice. **c** Quantitative RT–PCR confirmed that all transgenic lines express human *PRKCG* which encode PKCγ protein. **d** Purkinje cells in organotypic slice cultures from each transgenic mouse line are shown. Anti-calbindin staining showing all Purkinje cells and anti-GFP immunostaining is shown for transgene expression. Scale bar in H = 50 μm. **e** Each Purkinje cell area was calculated with image J and analyzed with Graphpad prism. Data are shown as the mean ± S.D. of at least 20 Purkinje cells ****p < 0.0001

In a previous study, we have reported that transgenic mouse line which carry the catalytic domain mutation S361G of PKCγ (here named PKC-C, Fig. 1a) caused severe inhibition of Purkinje cell dendritic development in organotypic cerebellar slice cultures [16]. The observed Purkinje cell morphology is identical to that of Purkinje cells treated with the PKC activator PMA indicating that the S361G mutation has increased kinase activity. We have generated two additional transgenic mouse lines which carry the G118D mutation located in the regulatory C1b domain (named PKC-A, Fig. 1a) or a partial deletion (Δ260–280) of the C2 domain (named PKC-B, Fig. 1a). A similar deletion was shown previously to confer an increased catalytic activity which was independent of PMA stimulation [21]. The total PKCγ protein expression level in each transgenic mouse was analyzed by Western blot and found to be well increased in PKC-C, less so in PKC-A and only slightly for PKC-B (Fig. 1b). In order to analyze transgene expression, qPCR was performed using human *PRKCG* specific primers and mouse *Prkcg* specific primers. While control mice expressed only mouse *Prkcg*, transgenic mice expressed both human *PRKCG* and mouse *Prkcg* (Fig. 1c). In contrast to the findings with PKC-C, Purkinje cell dendritic development was virtually normal in organotypic slice cultures derived from PKC-A or PKC-B mice (Fig. 1d). The findings suggest that the S361G mutation, but not the G118D mutation or the Δ260–280 deletion confer increased PKCγ biological activity to Purkinje cells.

### Transgenic mice carrying the S361G mutation but not G118D mutation or Δ260–280 showed inhibition of dendritic development in dissociated cerebellar culture

**Fig. 2:**
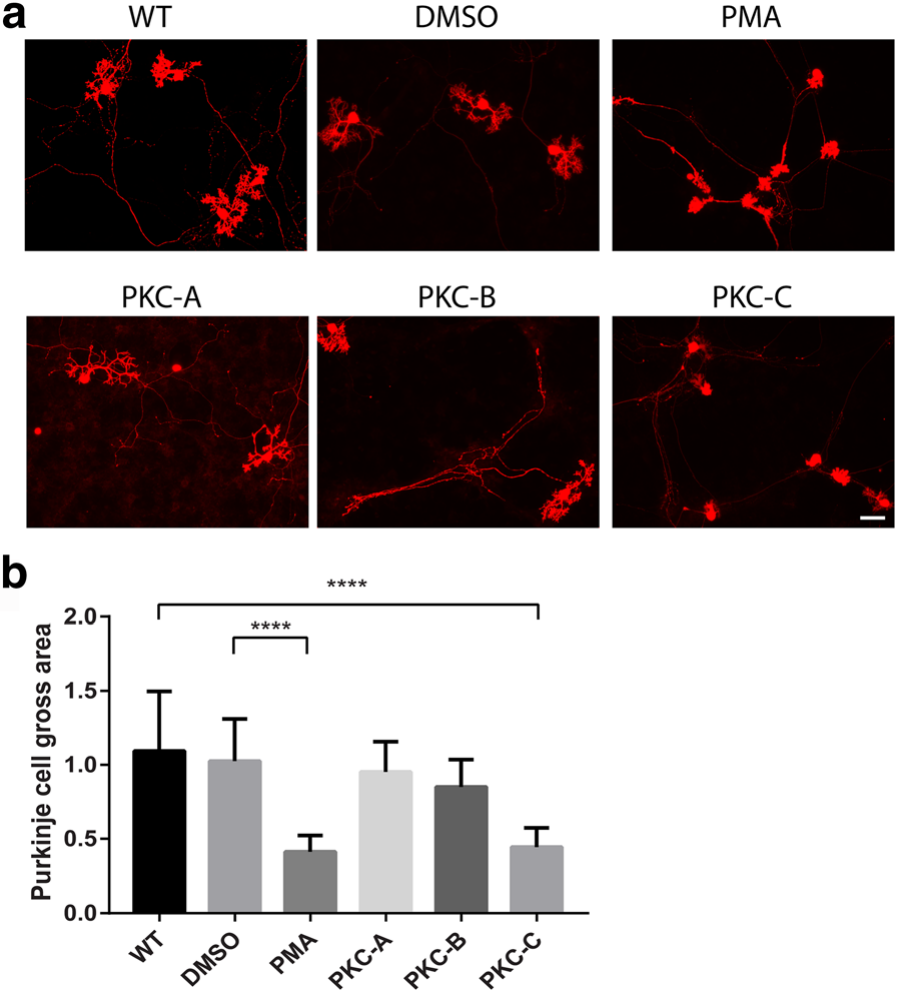
Morphology of Purkinje cells from PKCγ mutated transgenic mice in dissociated primary culture. The morphology of Purkinje cells was analyzed after 2 weeks in culture. **a** Wildtype (WT) Purkinje cells showed normal dendritic expansion in dissociated primary culture. DMSO treated Purkinje cells showed normal dendritic growth. PMA (15 nM) treated Purkinje cells showed inhibition of dendritic growth. Purkinje cells from PKC-A transgenic mice showed normal dendritic growth. Purkinje cells from PKC-B transgenic mice showed normal dendritic growth. Purkinje cells from PKC-C transgenic mice showed inhibition of dendritic growth. Scale bar = 50 μm. **b** Quantification of the Purkinje cell area. The dendritic tree size of Purkinje cells in PMA treated cultures or cultures from S361G transgenic mice was strongly reduced. Each Purkinje cell area was calculated with image J and analyzed with Graphpad prism. Data are shown as the mean ± S.D. of at least 20 Purkinje cells ****p < 0.0001

Organotypic cerebellar slice cultures are started at P8 and cultured for 1 week [19]. Dissociated cerebellar cultures are started at P0 and cultured for 2 weeks or more [18]. In order to confirm the results from organotypic slice cultures, we also did dissociated cerebellar cultures from the different PKCγ transgenic mouse models. Purkinje cells from PKC-C showed small dendritic trees with strongly reduced side branches similar to WT Purkinje cells treated with the PKC activator PMA (Fig. 2). We did the PMA treatment either from P4-P11 or from P7-P14, in both conditions Purkinje cells showed a similar morphology (data not shown). This finding indicated that PKC stimulation at an early stage (first postnatal week) or at a later stage (second postnatal week) induces morphological changes of Purkinje cell dendrites. In contrast, Purkinje cells from transgenic mice carrying the G118D mutation (PKC-A) or the deletion mutant Δ260–280 (PKC-B) didn’t show dendritic changes in dissociated cerebellar cultures (Fig. 2). These results confirm the findings from the organotypic cerebellar slice culture. Nevertheless, parts of the observed differences might be explained by different expression levels of mutant PKCγ in the different mouse models. In order to get a more complete picture about which type of mutations may induce dendritic changes in Purkinje cells, we tested diverse mutations from SCA14 patients in a transfection paradigm using PKCγ constructs under the L7 promoter transfected to Purkinje cells in dissociated cerebellar cultures.

### Choice of SCA14 mutations for expression in Purkinje cells

**Fig. 3:**
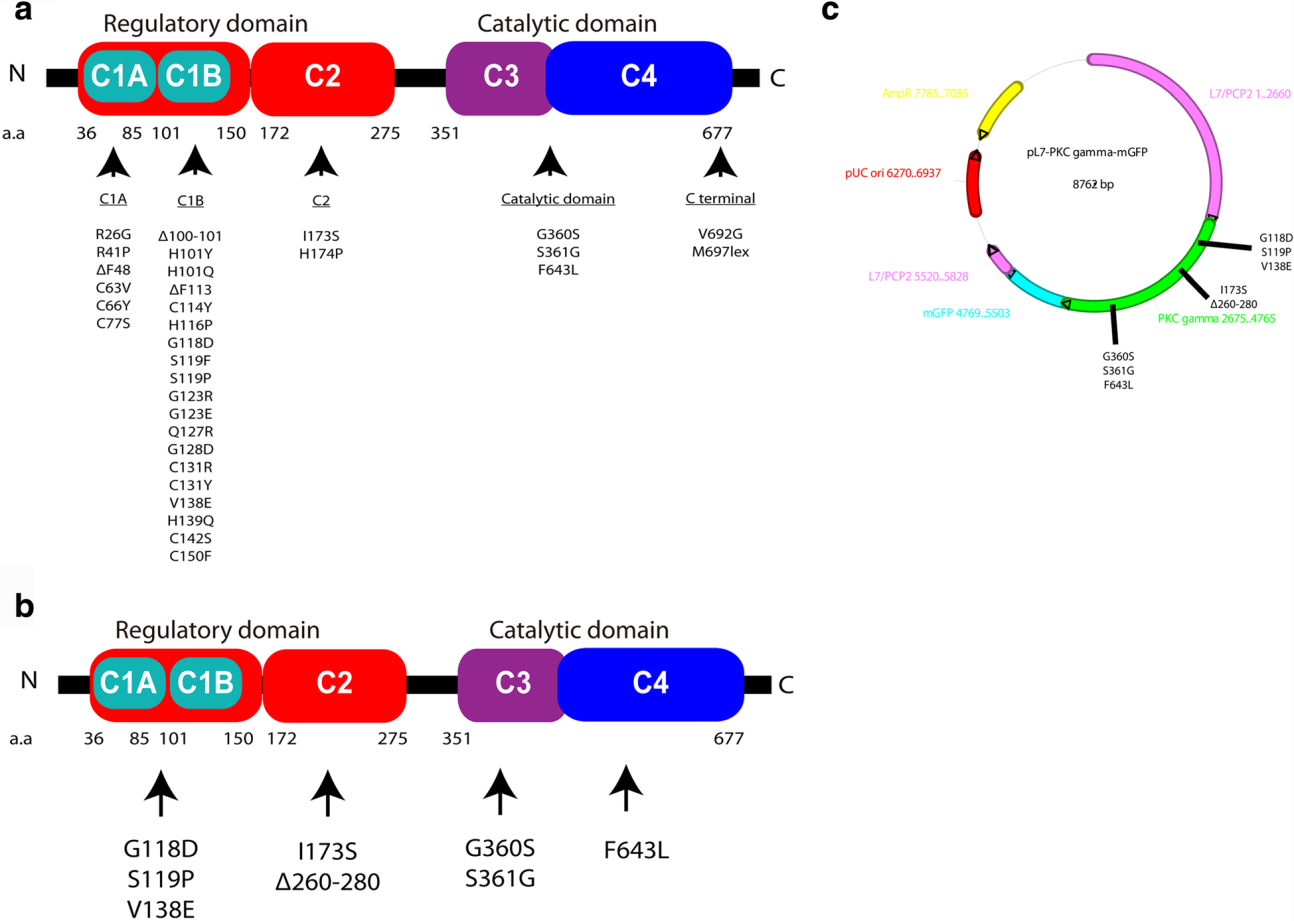
Illustration of PKCγ protein domain mutations and constructs for the experiments, **a** Map of 32 point mutations and deletions found in SCA14 families. Most mutations are found in the C1B domain. **b** Map of the seven point mutations and one deletion used in the transfection experiments in this study. **c** Vector map of the L7 expression vector used for Purkinje-cell specific expression of the transgenes. Each mutation or deletion was constructed as a GFP fusion protein

To date, over 30 mutations in PKCγ have been found in SCA 14 families (Fig. 3a). Many of them are concentrated on the C1B domain while other mutations are found throughout the coding regions of the *PRKCG* gene. We have constructed plasmids for three of the C1B mutations, one C2 mutation plus the deletion mutant Δ260–280 in the C2 domain which is not an SCA14 mutation but might be a constitutive active form [21] and the three known catalytic domain mutations (Fig. 3b). The L7 based plasmids yielded a Purkinje cell specific expression of the mutated proteins [18, 22, 23]. In order to confirm the transfection and expression of Purkinje cells easily, the PKCγ mutations were constructed as GFP fusion proteins (Fig. 3c).

### Purkinje cells expressing PKCγ with C1 or C2 domain mutations showed normal dendritic development

**Fig. 4:**
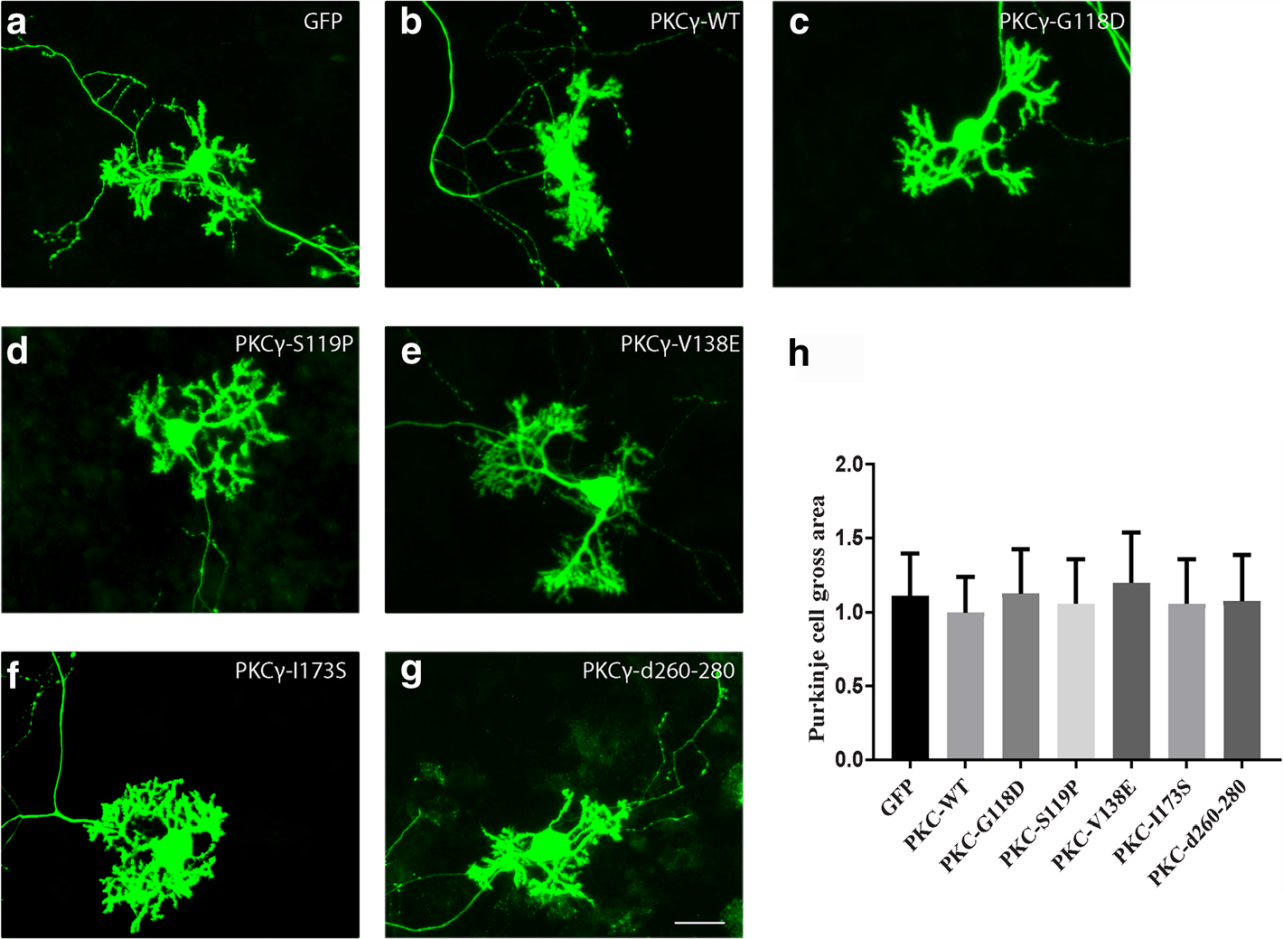
Morphology of Purkinje cells transfected with PKCγ C1 or C2 domain mutants. The morphology of Purkinje cells was analyzed after 2 weeks in culture. **a** Purkinje cells transfected with GFP control vector. **b** Purkinje cells transfected with PKCγ WT. **c** Purkinje cells transfected with PKCγ-G118D, **d** PKCγ-S119P, **e** PKCγ-V138E or **f** PKCγ-I173S. **g** Purkinje cells transfected with PKCγ Δ260–280. Scale bar in G = 50 μm. **h** Quantification of the Purkinje cell area. The dendritic tree size of Purkinje cells transfected with C1 or C2 domain mutations was not significantly different from control Purkinje cell transfected with the empty vector expressing GFP only or from PKCγ WT transfected Purkinje cells. Data are shown as the mean ± S.D. of at least 40 Purkinje cells. The value for Purkinje cells transfected with PKCγ WT was set as 100%

In order to exclude unspecific effects due to the transfection procedure and the culture environment findings were compared to Purkinje cells transfected with the same L7 expression plasmid only carrying a GFP control in the same experiment. In order to see the effect of transfection of wild type PKCγ (PKCγ WT) on Purkinje cell morphology, a GFP-PKCγ WT fusion plasmid was transfected to dissociated cerebellar cells. As a result of PKCγ WT expression, we found a slight but not statistically significant reduction of dendritic growth in PKCγ WT transfected Purkinje cells (Fig. 4a, b). This finding indicates that overabundance of WT PKCγ itself does not have a major inhibitory effect on dendritic tree development in Purkinje cells in dissociated cultures. We then did transfections with three of C1B mutations (G118D, S119P and V138E) which were reported as causing SCA14. Purkinje cells transfected with PKCγ carrying these mutations showed rather normal dendritic development (Fig.4c–e) and their morphology was virtually identical to that of Purkinje cells transfected with the GFP control plasmid. Statistical analysis showed no change of the area covered by the dendritic tree of the Purkinje cells transfected with plasmids carrying C1b mutations (Fig. 4h). The C2 domain mutation, L173S, is also causing SCA14 and the deletion mutant Δ260–280 located in the C2 domain was shown to induce increased activity in PKCα [21]. Purkinje cells transfected with PKCγ plasmids carrying either of these mutations also showed normal dendritic development (Fig. 4f, g). Statistical analysis showed no significant differences of the dendritic area of Purkinje cells transfected with the C2 domain mutation or the Δ260–280 deletion compared to GFP transfected Purkinje cells (Fig. 4h) indicating that the mutations in the C1b or C2 domain do not affect Purkinje cell dendritic development.

### Purkinje cells expressing PKCγ with two catalytic domain mutations showed inhibition of dendritic development

**Fig. 5:**
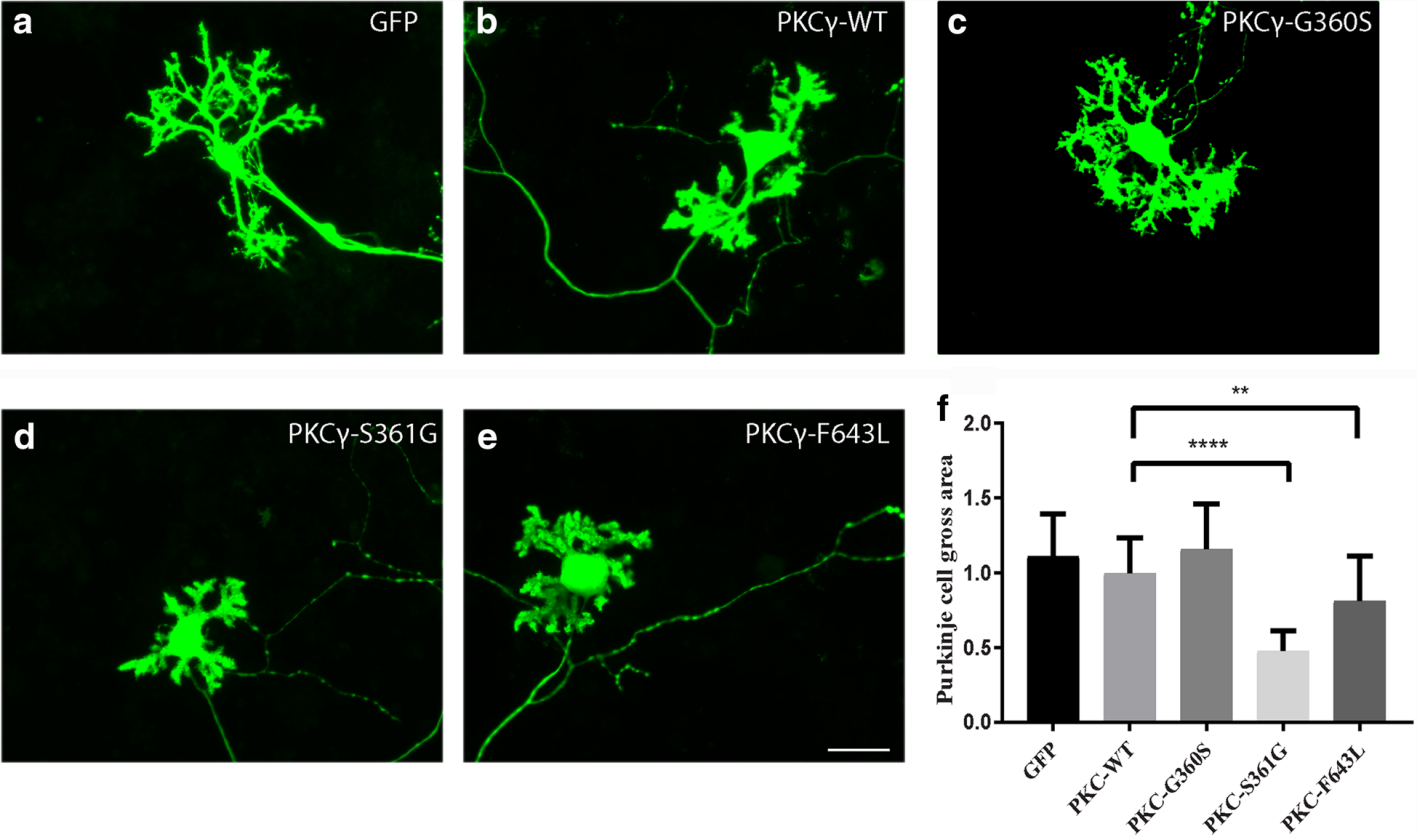
Morphology of Purkinje cells transfected with PKCγ catalytic domain mutants. The morphology of Purkinje cells was analyzed after 2 weeks in culture. **a** Purkinje cells transfected with GFP control vector. **b** Purkinje cells transfected with PKCγ WT. **c** Purkinje cells transfected with PKCγ-G360S. **d** Purkinje cells transfected with the PKCγ-S361G (**e**) Purkinje cells transfected with PKCγ-F643 L. Scale bar in E = 50 μm. **f** Quantification of the Purkinje cell area. The dendritic tree size of Purkinje transfected with the catalytic domain mutations S361G (*P* < 0.0001) or F643 L (*P* < 0.0025) was significantly reduced. Data are shown as the mean ± S.D. of at least 40 Purkinje cells. The value for Purkinje cells transfected with PKCγ WT was set as 100%

We have tested three mutations located in the catalytic domain, all of which were reported to cause SCA14. The S361G mutation was also used for constructing transgenic mice and was shown there and in the data above to confer increased PKCγ activity to Purkinje cells [16]. The F643 L mutation is also located in the catalytic domain and was shown to have increased kinase activity [6]. In contrast, the G360S mutation was reported to have a decreased or even absent catalytic activity [6]. Purkinje cells transfected with PKCγ carrying the G360S mutation showed rather normal dendritic development (Fig. 5c) similar to control Purkinje cells transfected with a GFP plasmid (Fig. 5a) or with a PKCγ WT plasmid (Fig. 5b).

In contrast, Purkinje cells transfected with PKCγ carrying the S361G mutation only developed small dendritic trees with few side branches (Fig.5d). This morphology is identical to that of Purkinje cells from PKCγ-S361G transgenic mice (Fig. 2). Similar results were obtained for the F643 L mutation. Transfected Purkinje cells showed inhibition of dendritic development (Fig. 5e) compared to GFP or PKCγ WT transfected Purkinje cells (Fig. 5a, b) although the effect was less pronounced as in S361G transfected Purkinje cells (Fig. 5d). Statistical analysis showed a reduction of the dendritic area of Purkinje cells transfected with the S361G mutation or the F643 L mutation compared to GFP or PKCγ WT transfected Purkinje cells (Fig. 5f, p<0.005). These results indicate that two catalytic domain mutations which are known to have higher PKCγ activity compared to PKCγ WT negatively regulate dendritic growth of Purkinje cells.

## Discussion

### PKCγ transgenic mice and mutant PKCγ kinase activity

We had reported earlier that transgenic mice with the PKCγ mutation S361G [16] show mild ataxia and strong abnormalities of dendritic growth of Purkinje cells in cerebellar slice culture [16, 24] and also in dissociated cerebellar culture [17]. We now tested two new transgenic mouse lines which Purkinje cell specific expression of PKCγ with the G118D mutation in the C1 domain or a deletion mutant Δ260–280 in the C2 domain. In organotypic slice cultures from both lines Purkinje cells showed normal dendritic development although the transgene was expressed as evident by GFP expression from the bidirectional vector. The expression of the human *PRKCG* transgenes was confirmed by qPCR. In order to analyze PKCγ protein expression levels in these lines we did Western blot analysis (Fig. 1) and found that PKCγ expression in PKC-A was approximately 140% of control mice, PKC-B was only approximately 120% of control mice, much less compared to approx. 270% expression in S361G mice compared to control mice. We don’t know the reason for the low expression in PKC-B and PKC-A mice. One possibility is that the protein carrying the deletion mutant Δ260–280 is subject to accelerated degradation, alternatively protein translation of the transcript might be compromised. Therefore, we cannot exclude that transgene expression in the G118D and Δ260–280 transgenic mice was too low to produce a phenotype.

### Similar results in organotypic slice cultures and dissociated cerebellar cultures

In our experiments we could essentially replicate the findings about Purkinje cell dendritic morphology from slice cultures of transgenic mice in dissociated cerebellar cultures. This is not trivial because cerebellar slice cultures cover the developmental period between P8 and P15 and Purkinje cells develop in a virtually intact microenvironment [25, 26]. In contrast, dissociated cerebellar cultures are established from P0 mice and are maintained for about 15 days. In contrast to slice cultures, in dissociated cultures Purkinje cells are more isolated and have only limited contact to other cerebellar cells which, however, are all present in these cultures [27]. The Purkinje cells at the end of the culture period appear more immature compared Purkinje cells in slice cultures and probably rather correspond to a stage around P10. Despite these important differences, the major outcome with respect to PKC activity and dendritic morphology is very similar and comparable [17]. This makes it very likely that the increased PKC activity works in a cell autonomous way and that the effect is robust enough for not being modified by different culture conditions or cell-cell-interactions. It confirms that Purkinje cell transfections in dissociated cerebellar cultures are a valid tool for analyzing the effects of various genes for Purkinje cell dendritic development.

### SCA14 related PKCγ mutations and mutant PKCγ kinase activity

In order to address whether mutations of PKCγ would affect biological activity, we turned to transfection of Purkinje cells with plasmids of mutant PKCγ in dissociated cerebellar cultures. This did also allow us to test more mutations associated with SCA14. It should be noted that with this assay the endogenous PKCγ protein is still in place making the assay suitable for detecting an increased biological activity but not for detecting a loss of activity which could only be indirectly assessed by comparing transfection of the mutated protein to transfection of the WT protein. Using this system, we found that the overexpression of PKCγ WT only induced a slight reduction of Purkinje cells dendritic tree size. This is in agreement with previous findings that PKCγ has a negative effect on Purkinje cell dendritic growth.

When we tested the mutations from the transgenic mouse lines we found that the PKCγ-S361G was again very potent in inhibiting Purkinje cell dendritic growth, in contrast, transfection with the PKCγ-G118D and PKCγ-Δ260–280 resulted in virtual normal dendritic growth. Dendritic development after transfection with these constructs was clearly superior to that seen after PKCγ-S361G transfection suggesting that the biological activity of these mutants in Purkinje cells was normal or even reduced.

We made constructs for two more SCA14 mutations located in the C1B domain, the S119P and the V138E mutation. In both cases transfection resulted in normal dendritic development of Purkinje cells. Taken together, neither in the G118D transgenic mouse line nor in any of three independent C1B domain mutations showed any evidence for a negative effect for dendritic growth, rather there is some evidence that the biological activity of these mutations might be reduced because dendritic development was somewhat superior (though not statistically significant different) compared to transfection of PKCγ WT. This makes it very unlikely that an increase of PKC activity is involved in the pathogenesis of the C1B mutations. This finding is in agreement with reports finding rather a loss of biological activity of C1B mutations [6, 13, 28]. We got similar results for mutations in the C2 domain. The Δ260–280 PKCγ mutation was introduced with the idea that a similar deletion induced increased constitutive PKC activity in PKCα [21]. However, neither this deletion, either in the transgenic mouse line or after transfection of an expression plasmid, nor the I173S mutation located in the C2 domain had any negative effect on Purkinje cell dendritic growth suggesting that also the C2 domain mutations are associated with normal or even with a reduced biological PKC activity. This situation was rather different for the three reported SCA14 mutations in the catalytic domain of PKCγ. One of these mutations, the G360S, has already been reported to have a reduced catalytic activity (“kinase dead”, [6]). PKCγ carrying this mutation, when transfected, had no effect on Purkinje cell dendritic development (Fig. 5). Quite in contrast, we had shown previously high biological activity for the S361G mutation in the PKC-C and after transfection to Purkinje cells in dissociated cerebellar cultures [17] which was confirmed in this study. When we transfected plasmids with PKCγ carrying the catalytic domain mutation F643 L, we found reduction of dendritic growth as with S361G although the effect was weaker than for PKCγ-S361G. This shows that catalytic domain mutations with increased activity in cell based assays also have an increased biological activity in Purkinje cells.

### Biological activity of mutant PKCγ and the pathogenesis of SCA14

The finding, that 2 out of 3 mutations of SCA14 in the catalytic domain do indeed confer an increased biological activity of PKCγ of course suggest that this increased activity may be involved in the pathogenesis of SCA14. The increased biological activity of catalytic domain mutations is in contrast to the mutations in the C1B or in the C2 domain. These mutations were also shown to have an increased activity in cell based in vitro assays very similar to that of the kinase domain mutations [6, 11], but they failed to show any increased biological activity in Purkinje cells after transfection. This suggests that the mutations in the regulatory C1B and C2 domains in Purkinje cells will eventually lead to PKC variants with decreased function, probably due to mechanism like increased autophosphorylation, inefficient activation of signaling targets or aggregation as reported earlier [6, 10, 12, 13]. In contrast, the catalytic domain mutations obviously make the catalytic domain active irrespective of any effects of the regulatory domains and eventually there will be increased biological activity after transfection to Purkinje cells. While there is this clear distinction between the biological activities of regulatory domain vs catalytic domain mutations, both will eventually cause SCA14 with rather similar clinical and pathological findings. The pathological mechanisms leading to disease therefore are probably not linked in a simple way to the biological activity of the mutant PKCγ. One possibility might be that not the activity itself but a “loss of regulation” is an important factor in pathogenesis. Neither regulatory domain nor catalytic domain mutations will be able to adjust their activity rapidly to the needs of the Purkinje cell which will be leading to inappropriate kinase activity in many situations eventually leading to Purkinje cell dysfunction and death. Another possibility would be that pathogenesis is different for different types of mutations, with the increased PKCγ activity being an important factor in catalytic domain mutations, but with other mechanisms being active in regulatory domain mutations. It is also possible that the point mutations do affect biological activity differently, but this may not be the crucial aspect for the development of the disease, which could e.g. be promoted by protein aggregation [14, 15] of other mechanisms [29]. Taken together, our findings show, that biological activity of PKCγ is affected in opposing ways for catalytic versus regulatory domain mutations but the reasons for the similar neuropathological changes in both conditions still remain elusive.

## Abbreviations

Ca: Calcium
CB6: B6CF1, mouse strain
DAG: Diacylglycerol
DFM: Dulbecco’s modified eagle medium:F-12 nutrientbased medium
DIV: Days in vitro
P: Postnatal day
PB: Phosphate buffer
PKC: Protein kinase C
PMA: Phorbol-12-myristate-13-acetate
SCA: Spinocerebellar ataxia
